# Improvement in Functional Outcome from 6 to 12 Months After Moderate and Severe Traumatic Brain Injury Is Frequent, But May Not Be Detected With the Glasgow Outcome Scale Extended

**DOI:** 10.1089/neur.2023.0109

**Published:** 2024-02-29

**Authors:** Rabea Iris Pantelatos, Jonas Stenberg, Turid Follestad, Oddrun Sandrød, Cathrine Elisabeth Einarsen, Anne Vik, Toril Skandsen

**Affiliations:** ^1^ Department of Neuromedicine, Movement Science, and Faculty of Medicine and Health Sciences, Norwegian University of Science and Technology, Trondheim, Norway.; ^2^ Department of Clinical Sciences, Danderyd Hospital, Division of Rehabilitation Medicine, Karolinska Institutet, Stockholm, Sweden.; ^3^ Department of Radiology and Nuclear Medicine, Department of Neurosurgery, St. Olavs Hospital, Trondheim University Hospital, Trondheim, Norway.; ^4^ Clinical Research Unit Central Norway, Department of Neurosurgery, St. Olavs Hospital, Trondheim University Hospital, Trondheim, Norway.; ^5^ Department of Clinical and Molecular Medicine, Faculty of Medicine and Health Sciences, Norwegian University of Science and Technology, Trondheim, Norway.; ^6^ Clinic of Anaesthesia and Intensive Care, Department of Intensive Care Medicine, Department of Neurosurgery, St. Olavs Hospital, Trondheim University Hospital, Trondheim, Norway.; ^7^ Clinic of Rehabilitation, Department of Neurosurgery, St. Olavs Hospital, Trondheim University Hospital, Trondheim, Norway.; ^8^ Neuroclinic, Department of Neurosurgery, St. Olavs Hospital, Trondheim University Hospital, Trondheim, Norway.

**Keywords:** adult, clinical deterioration, cohort studies, craniocerebral trauma, Disability Rating Scale, Glasgow Coma Scale, Glasgow Outcome Scale, longitudinal studies, patient outcome assessment, prospective studies, recovery of function

## Abstract

The aims of this study were (1) to report outcome and change in outcome in patients with moderate and severe traumatic brain injury (mo/sTBI) between 6 and 12 months post-injury as measured by the Glasgow Outcome Scale Extended (GOSE), (2) to explore if demographic/injury-related variables can predict improvement in GOSE score, and (3) to investigate rate of improvement in Disability Rating Scale (DRS) score, in patients with a stable GOSE. All surviving patients ≥16 years of age who were admitted with mo/sTBI (Glasgow Coma Scale [GCS] score ≤13) to the regional trauma center in Central Norway between 2004 and 2019 were prospectively included (*n* = 439 out of 503 eligible). GOSE and DRS were used to assess outcome. Twelve-months post-injury, 13% with moTBI had severe disability (GOSE 2–4) versus 27% in sTBI, 26% had moderate disability (GOSE 5–6) versus 41% in sTBI and 62% had good recovery (GOSE 7–8) versus 31% in sTBI. From 6 to 12 months post-injury, 27% with moTBI and 32% with sTBI had an improvement, whereas 6% with moTBI and 6% with sTBI had a deterioration in GOSE score. Younger age and higher GCS score were associated with improved GOSE score. Improvement was least frequent for patients with a GOSE score of 3 at 6 months. In patients with a stable GOSE score of 3, an improvement in DRS score was observed in 22 (46%) patients. In conclusion, two thirds and one third of patients with mo/sTBI, respectively, had a good recovery. Importantly, change, mostly improvement, in GOSE score between 6 and 12 months was frequent and argues against the use of 6 months outcome as a time end-point in research. The GOSE does, however, not seem to be sensitive to actual change in function in the lower categories and a combination of outcome measures may be needed to describe the consequences after TBI.

## Introduction

Patients who survive from a moderate or severe traumatic brain injury (mo/sTBI) often experience physical, cognitive, and emotional problems.^1^ This represents a huge burden, not only for the patients, but also for their families and the society.^2^ The observed functional outcomes after survived mo/sTBI span from complete recovery to persistent vegetative state and reported rates of disability vary between settings and countries. Most studies reporting 12-month functional outcome include only sTBI^3–9^ or do not distinguish between moTBI and sTBI.^10,11^ Because the case fatality proportions in sTBI are high,^12–14^ the deceased patients will strongly influence the reported proportions of patients with poor outcome if they are not excluded in studies of outcome.^15^ In the clinical setting, however, the question of future functioning is typically raised in surviving patients, and studies are needed that explore outcome specifically among these.

Although many studies report 12-month functional outcome,^4–10,15–20^ the 6-month outcome is still used as an end-point in large studies,^21–23^ and is often used in prognostic models.^24,25^ However, patients, families, and clinicians will often observe meaningful improvement in functional ability also beyond the first 6 months. It is therefore of interest to quantify the amount of change in functional outcome, occurring between 6 and 12 months after surviving mo/sTBI. Changes in functional outcome have been investigated mostly in sTBI and with large differences in results.^4,9,26,27^ Few studies have explored characteristics of patients who show functional recovery between 6 and 12 months.^26,28^ A previous study reported that higher Glasgow Coma Scale (GCS) score and Traumatic Coma Data Bank Computed Tomography (CT) classification (I, II, and V) was associated with improved outcome.^28^ Knowledge of patients who will likely experience a protracted functional recovery is useful when planning rehabilitation and follow-up.

The Glasgow Outcome Scale Extended (GOSE) is, to our knowledge, the most used measure to assess functional outcome after TBI. Yet, it has faced criticism in the past for being too simplistic.^1,30^ The Disability Rating Scale (DRS) is considered the main alternative to the GOSE,^31^ and seems to be more fine-graded, especially regarding elementary abilities in daily life.^32^ The DRS may therefore be well suited to investigate the ability of the GOSE to capture change in function.

In this 15-year inception cohort study of mo/sTBI at a level I/II trauma center in Norway, the aims were to (1) report outcome and change in outcome between 6 and 12 months as measured by the GOSE, (2) to explore if demographic/injury-related variables can predict improvement in GOSE score, and (3) to investigate the proportion who experienced improvement in DRS score, despite a stable GOSE.

## Methods

### Setting and study period

This study utilize data from the Trondheim Moderate and Severe TBI study, an on-going inception cohort study conducted at St. Olavs Hospital, Trondheim University Hospital.^33^ The St. Olavs hospital is a level I/II trauma center and is also the regional referral center for all neurosurgical procedures in Central Norway, a region covering two counties with a total population of 729,452 inhabitants (2019). Data in the current study were collected between October 1, 2004 and October 1, 2019.

### Participants

**FIG. 1.**
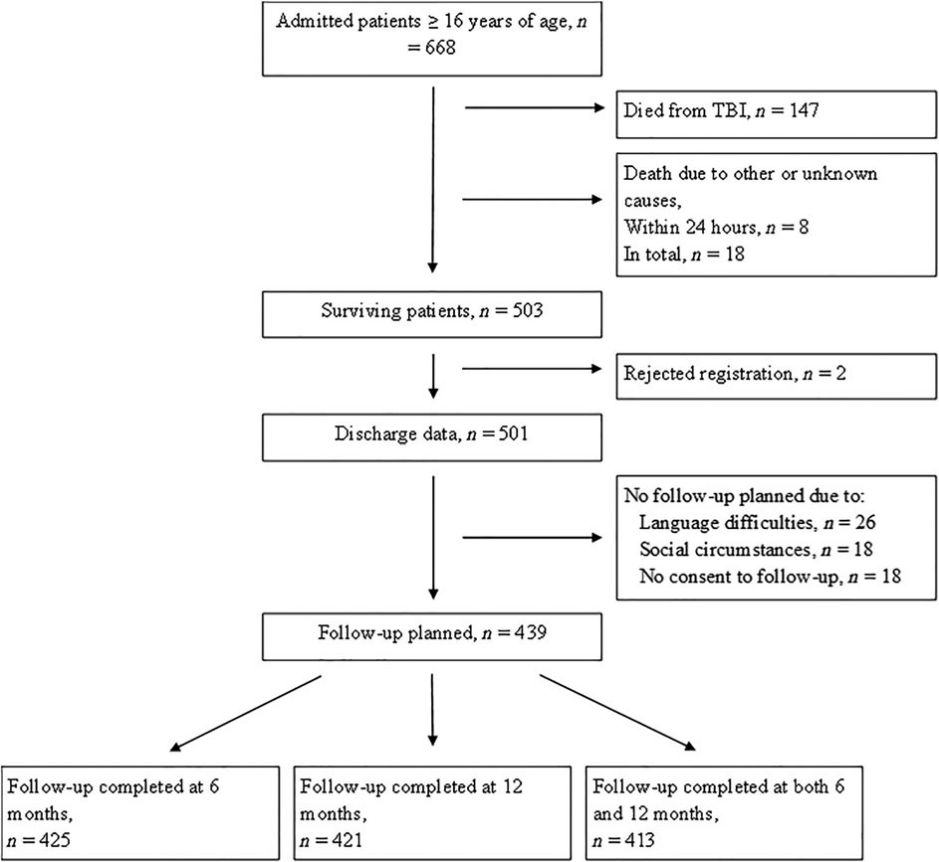
The inclusion of surviving patients with moderate and severe traumatic brain injury admitted to the St. Olavs hospital.

All patients with mo/sTBI admitted to St. Olavs Hospital within 72 h of the time of injury are included in the Trondheim Moderate and Severe TBI study. MoTBI is defined by a GCS score of 9–13, and sTBI is defined by a GCS score ≤8. Patients who die from extracranial injuries within the first 24 h after injury are excluded; otherwise, there are no exclusion criteria. All patients are planned for follow-up, except patients living in foreign countries, patients not able to speak a Scandinavian language or English, and patients with severe psychosocial strains not compatible with follow-up routines. In the current study, only patients ≥16 years of age, who were alive at 6 months after their TBI and planned for follow-up were included (Fig. 1).

### Study procedures

Patients were identified by residents in neurosurgery or a study nurse and were prospectively included. Additionally, the hospital's trauma registry was retrospectively checked regularly. Since 2015, a study nurse has been screening head CT referrals weekly. Acute phase and discharge data were mostly prospectively obtained from medical records. The 6-month and 12-month follow-ups were conducted at visits or by phone by trained interviewers. In cases in which the patients' self-reported outcome could be influenced by cognitive impairments or lack of awareness, a close relative or caregiver was also interviewed (in 51% of cases), when possible with the consent of the patient.

### Study variables

Demographic variables were age and biological sex (male, female). Work/study status at the time of injury was registered as *yes* if the patient had a paid position or was registered as a student. Education was categorized as primary and lower secondary school, upper secondary school, and college/university.

Comorbidity was defined as *yes* if severe enough to influence daily functioning, and if *yes,* it was categorized as substance abuse, psychiatric disorder, neurological disease, heart/lung disease, developmental disorder, cancer, several diagnoses, or other.

Injury-related variables were external cause of injury (traffic accident, fall, violence, gunshot injury, other, unknown) and GCS score obtained at admission to the trauma center or at the first hospital when transfer time was >6 h. In cases of pre-hospital intubation, the last non-sedated GCS score was used. In cases of severe intoxication or sedation, the GCS score was considered unreliable and handled as missing data. Head CT scans were reviewed for research purposes by a resident or consultant in neurosurgery or radiology and blinded for patient outcome, and findings on the worst scan were reported.

Specialized rehabilitation was here defined as rehabilitation delivered in one of the three hospital-based departments of rehabilitation in the region. These departments provide in-hospital and out-patient multidisciplinary rehabilitation services. The data on rehabilitation were registered retrospectively by review of hospital patient records and were categorized as unknown in patients transferred to further acute care in other health regions in Norway and in patients with injuries that required other excessive rehabilitation (i.e. spinal cord injury).

TBI-related functional outcome was assessed with the GOSE,^34^ (Table 1). Severe disability was defined as GOSE scores 3–4, moderate disability was defined as GOSE scores 5–6 and good recovery was defined as GOSE scores 7–8. The DRS was used as a measurement of global functional outcome and was not TBI specific. The DRS ranges from 0 to 30 (death).^32^ A score of 0 indicates no disability and a score of 29 represents deep coma. The scale consists of eight items, where the first three items are derived from the GCS with an assessment of eye opening, verbal communication, and motor response. Further items assess the cognitive ability for self-care (feeding, toileting, and grooming), level of independence, and employability.^32^

**Table 1.** The Glasgow Outcome Scale Extended^a^

***Glasgow Outcome Scale Extended Score***	***Interpretation***
8 = Upper good recovery	Fully recovered or may have minor symptoms not affecting daily life
7 = Lower good recovery	Able to return to previous life roles, but with symptoms that affect daily life
6 = Upper moderate disability	Some disability exists, but able to partly return to previous life roles
5 = Lower moderate disability	Independent, but cannot return to one or more life roles
4 = Upper severe disability	Dependent, needs infrequent assistance in basic activities in daily life, or help with activities outside the home
3 = Lower severe disability	Dependent, needs frequent assistance in basic activities in daily life
2 = Vegetative state	No awareness of self or environment
1 = Dead	Dead

^a^ The Glasgow Outcome Scale Extended^34,56^ is an extension of the Glasgow Outcome Scale evolved by Jennet and Bond in 1975.^54^ Table 1 is modified from the thesis of Olsen.^35^

### Statistical analysis

The data were presented as mean, median, or percentages, depending on distribution. Change in functional outcome was calculated by subtracting the GOSE score at 6 months from the GOSE score at 12 months. The change was categorized as improvement, stable, or deterioration. The associations between demographic/injury-related variables and change in GOSE score (improvement, no improvement) were explored with univariable and multivariable binary logistical regression analyses. In these analyses, the GCS score was chosen as the variable reflecting injury severity because the score is affected not only by large, focal lesions causing increased intracranial pressure, but also by the presence of traumatic axonal injuries, which may not be detected by CT.^36,37^ All patients with a GOSE score of 8 at 6 months were excluded from the univariable/multivariable logistical regression analyses because these patients could not improve further. A significance level of 0.05 was set.

Missing data were handled by pairwise deletion/complete case analysis, excluding patients with at least one missing observation in the outcome or explanatory variables (i.e., comorbidity, level of education, GCS score, and/or GOSE score at 6 and 12 months) from the logistical regression analyses.

The IBM SPSS version 28 software was used in all analyses.^38^

### Ethics

The study was approved by the Regional Committee for Medical Research Ethics (2009/2328). Consent was obtained from surviving patients eligible for follow-up, or for incapacitated patients, from their next of kin.

## Results

### Patient characteristics

Out of 439 surviving patients planned for follow-up, 57% had moTBI and 43% had sTBI. The mean age was 45 years and 74% were males. In 18% of the patients, pre-injury disabling health conditions were present at the time of injury. The most common external causes of injury were falls (47%) and traffic accidents (44%). Specialized rehabilitation was more frequent in patients <65 years of age (84%) than in patients ≥65 years of age (36%) (Table 2).

**Table 2.** Demographic and Injury-Related Characteristics of 439 Patients Included and Planned for Follow-Up

***Variable***	***All included patients*** *n* *** = 439***
Age at injury (years)	
Mean (SD)	45 (20)
Sex, *n* (%)	
Male	325 (74)
Female	114 (26)
Comorbidity,^a^ *n* (%)	
None	338 (82)
Substance abuse	32 (8)
Psychiatric disorders	14 (3)
Neurological disease	17 (4)
Heart/lung disease	≤ 3
Developmental disorder	≤ 3
Cancer	≤ 3
Several diagnoses	≤ 3
Other	≤ 3
Work/study status before injury,^b^ *n* (%)	
Yes	289 (68)
No	65 (15)
Retired	69 (16)
External cause of injury, *n* (%)	
Fall	200 (46)
Traffic accident	191 (44)
Violence	14 (3)
Other	24 (5)
Unknown	10 (2)
GCS score at admission and severity of TBI	
Median (IQR)^c^	9 [6, 13]
Moderate TBI (GCS score 9-13), *n* (%)	249 (57)
Severe TBI (GCS score ≤8), *n* (%)	190 (43)
CT findings, *n* (%)	
Subdural hemorrhage	230 (52)
Epidural hemorrhage	73 (17)
Subarachnoid hemorrhage (basal or cortical)	264 (60)
Intraventricular hemorrhage	89 (20)
Single contusion	63 (14)
Multiple contusions	209 (48)
General edema	81 (19)
Fracture (no impression)	217 (49)
Impression fracture	36 (8)
Intracranial air	117 (27)
Other	222 (51)
Specialized rehabilitation, *n* (%)	
Age <65 years	
Yes	267 (74)
No	52 (14)
Unknown	40 (11)
Age ≥65 years	
Yes	29 (36)
No	44 (55)
Unknown	7 (9)

^a^ In 27 patients presence of comorbidity was unknown. The percentages are calculated from 412 patients.

^b^ In 16 patients work/study status was unknown. The percentages are calculated from 423 patients.

^c^ In 19 patients it was not possible to determine a reliable GCS score at admission.

CT, computed tomography; GCS, Glasgow Coma Scale; IQR, interquartile range; SD, standard deviation; TBI, traumatic brain injury.

### Outcome in surviving patients

In moTBI, at 6 months 17% had a severe disability, 34% had a moderate disability, and 49% had a good recovery; at 12 months, 13% had a severe disability, 26% had a moderate disability, and 62% had a good recovery. In sTBI, at 6 months 31% had a severe disability, 49% had a moderate disability, and 20% had a good recovery; at 12 months, 27% had a severe disability, 41% had a moderate disability, and 31% had a good recovery. Most with severe disability were in the lower level of this category, and most with good recovery were in the upper level, both in moderate and severe TBI. Only two patients (1%) with sTBI were in a vegetative state 12 months after injury (Table 3).

**Table 3.** Global Function Measured With the Glasgow Outcome Scale Extended at 6 and 12 Months in Patients With Moderate and Severe Traumatic Brain Injury

	All TBI^*^		Moderate TBI^*^		Severe TBI^*^	
	***6 months***	***12 months***	***6 months***	***12 months***	***6 months***	***12 months***
***GOSE score***	***n* * = 425***	***n* * = 421***	***n* * = 242***	***n* * = 240***	***n* * = 183***	***n* * = 181***
1, *n* (%)	.	2 (0.5)	.	0	.	2 (1)
2, *n* (%)	4 (1)	2 (0.5)	0	0	4 (2)	2 (1)
3, *n* (%)	70 (16)	55 (13)	30 (13)	23 (10)	40 (22)	32 (18)
4, *n* (%)	21 (5)	20 (5)	9 (4)	7 (3)	12 (7)	13 (7)
5, *n* (%)	96 (23)	62 (15)	37 (15)	23 (10)	59 (33)	39 (22)
6, *n* (%)	74 (17)	70 (17)	45 (19)	36 (16)	29 (16)	34 (19)
7, *n* (%)	54 (13)	66 (16)	39 (16)	43 (19)	15 (8)	23 (13)
8, *n* (%)	100 (24)	133 (32)	79 (33)	100 (43)	21 (12)	33 (18)
No reliable score,^a^ *n*	6	6	3	3	3	3
Death from other cause,^a^ *n*	.	5	.	5	.	0

^*^ Percentages are calculated based on the number of non-missing values for each group and time point.

^a^ Not included in the percentages.

GOSE, Glasgow Outcome Scale Extended; TBI, traumatic brain injury.

### Change in GOSE score from 6 to 12 months

**FIG. 2.**
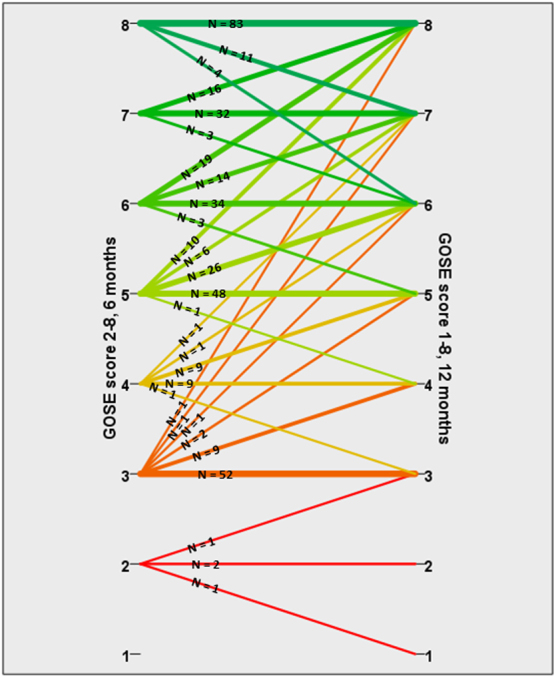
Parallel coordinates plot illustrating change in Glasgow Outcome Scale Extended (GOSE) score from 6 to 12 months in surviving patients with moderate and severe traumatic brain injury. Only patients with a reliable GOSE score at the 6- and 12-month follow-up, *n* = 401, are shown here.

Of all patients with TBI, 35% experienced a change in GOSE score after 6 months. Twenty-seven percent with moTBI and 32% with sTBI had an improvement, whereas 6% with mo/sTBI had a deterioration (Table 4). Figure 2 shows in detail the number of patients who improved, were stable or had deteriorated, depending on their GOSE score at 6 months. The percentage of improvement according to the 6-month GOSE score was as follows: of patients with a GOSE score of 3 at 6 months, 21% improved; of those with a GOSE score of 4, 52% improved; of those with a GOSE score of 5, 46% improved, of those with a GOSE score of 6, 47% improved, and of those with a GOSE score of 7, 31% improved. Of patients with a GOSE score of 8 at 6 months, 85% were stable at 12 months.

**Table 4.** Change (Improvement or Deterioration) in Glasgow Outcome Scale Extended Score from 6 to 12 Months in Surviving Patients With Moderate and Severe Traumatic Brain Injury

	***All TBI***	***Moderate TBI***	***Severe TBI***
***Change in outcome***	***n* * = 401^a^***	***n* * = 227^a^***	***n* * = 174^a^***
Improvement, *n* (%)	117 (29)	61 (27)	56 (32)
Stable, *n* (%)^b^	260 (65)	153 (67)	107 (61)
Stable 8	83 (32)	68 (44)	15 (14)
Stable 4-7	125 (48)	63 (41)	62 (58)
Stable 3	52 (20)	22 (14)	30 (28)
Deterioration, *n* (%)	24 (6)	13 (6)	11 (6)

^a^ Only surviving patients (at 6 months post-injury) with valid GOSE scores both at 6- and 12- month follow-up were included in these analyses.

^b^ Patients in the category “Stable 4-7” had the same GOSE score at 6 and 12 months (e.g., 4 to 4, 5 to 5).

GOSE, Glasgow Outcome Scale Extended; TBI, traumatic brain injury.

### Factors associated with improvement in GOSE score at 12 months

From the univariable analyses, higher age (odds ratio [OR] 0.98, 95% confidence interval [CI] 0.97–0.99, *p* = 0.004) and presence of comorbidity (OR 0.53, 95% CI 0.29–0.95, *p* = 0.034) was significantly negatively associated with improvement in GOSE score at 12 months (Table 5). From the multivariable analysis, higher age was significantly negatively associated with improvement in GOSE score (OR 0.98, 95% CI 0.96–0.99, *p* = 0.003) and higher GCS score at admission was positively associated with improvement in GOSE score (OR 1.08, 95% CI 1.00–1.17, *p* = 0.049) at 12 months. Presence of comorbidity was not a significant predictor of improvement in the multivariable analysis; however, the OR did not change much from the univariable analysis (OR 0.62, 95% CI 0.33–1.19, p = 0.152) (Table 5).

**Table 5.** Uni- and multivariable Logistic Regression Analyses of Change in Glasgow Outcome Scale Extended Score from 6 to 12 Months (Improved vs. Not Improved) on Demographic and Injury-Related Variables^a^

			***Crude***		***Adjusted^b^***	
	***Not improved***, ***n = 186***	***Improved***, ***n = 117***	***OR (95% CI)***	*p* ***value***	***OR (95% CI)***	p ***value***
Age (years)			0.98 (0.97-0.99)	0.004	0.98 (0.96-0.99)	0.003
Mean (SD)	48 (20)	42 (18)				
Median (IQR)	50 [30, 63]	42 [24, 55]				
Sex						
Male, *n* (%)	130 (70)	90 (77)	1.44 (0.84-2.45)	0.183	1.40 (0.78-2.52)	0.260
Level of education, *n* (%)	^c^	^d^				
Primary and lower secondary school	53 (30)	24 (21)	Ref.	0.214	Ref.	0.712
Upper secondary school	75 (43)	57 (50)	1.68 (0.93-3.04)	0.087	1.19 (0.63-2.28)	0.591
College/University	48 (27)	34 (29)	1.56 (0.82-3.00)	0.179	1.34 (0.67-2.71)	0.411
Comorbidity	^e^	^f^				
Yes, *n* (%)	50 (27)	19 (16)	0.53 (0.29-0.95)	0.034	0.62 (0.33-1.19)	0.152
Severity of TBI	^g^	^h^				
GCS score, median (IQR)	8 (5, 12)	9 (6, 12)	1.03 (0.97-1.10)	0.323	1.08 (1.00-1.17)	0.049

^a^ Only surviving patients (at 6 months post-injury) with valid GOSE score 2-7 at 6-month and valid GOSE score 1-8 at 12-month follow-ups were included in these analyses, *n* = 303.

^b^ The adjusted model had 22 missing cases. The model was adjusted for the following variables: age (years), sex, level of education, comorbidity, and GCS score.

^c^ In 10 patients education status was unknown. The percentages are calculated from 176 patients.

^d^ In 2 patients education status was unknown. The percentages are calculated from 115 patients.

^e^ In 1 patient presence of comorbidity was unknown. The percentages are calculated from 185 patients.

^f^ In 1 patient presence of comorbidity was unknown. The percentages are calculated from 116 patients.

^g^ In 5 patients no reliable GCS score could be determined. The median and interquartile ranges are calculated from 181 patients.

^h^ In 3 patients no reliable GCS score could be determined. The median and interquartile ranges are calculated from 114 patients.

CI, confidence interval; GCS, Glasgow Coma Scale. GOSE, Glasgow Outcome Scale Extended; IQR, interquartile range; OR, odds ratio; TBI, traumatic brain injury.

### Change in DRS scores when GOSE score was stable

**FIG. 3.**
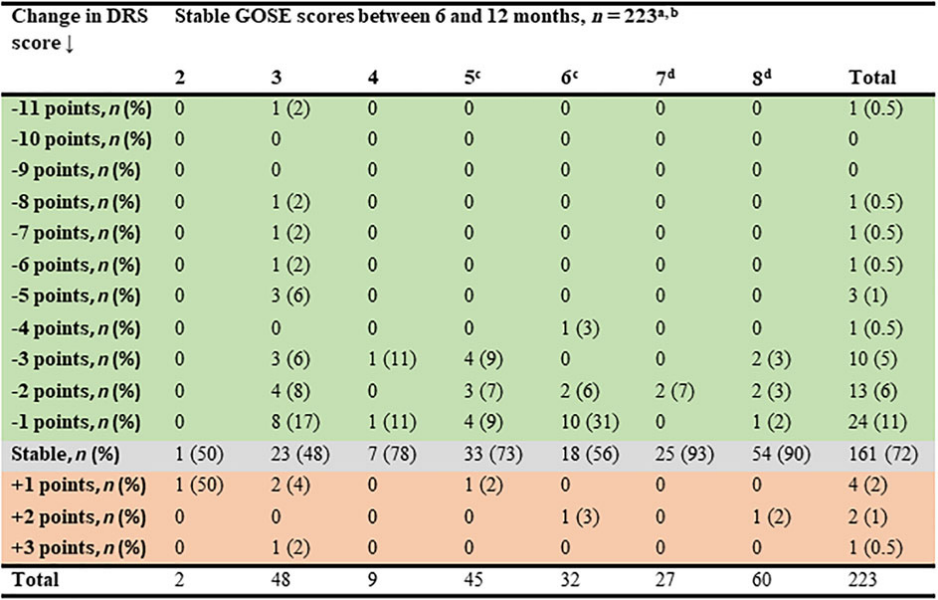
Change in Disability Rating Scale (DRS) score in patients with stable Glasgow Outcome Scale Extended (GOSE) score between 6- and 12-month follow-ups. ^a^Red color indicates deterioration, gray color indicates stable, and green color indicates improvement in DRS score. ^b^Only surviving patients (at 6 months post-injury) with no change in GOSE score between 6- and 12-month follow-ups (*n* = 260) and with a reliable DRS score were included in the percentages. Thirty-seven had missing DRS scores; therefore, the percentages are calculated from 223 patients. ^c^One patient with a stable GOSE score of 5 and three patients with a stable GOSE score of 6 had other disabilities/diseases influencing the total life situation assessed with the DRS score. ^d^Patients with a stable GOSE score of 7 or 8 with a change in DRS score (*n* = 8), were on sick leave at 6 months because of other injuries (not traumatic brain injury [TBI] related).

Of all patients with the same value of GOSE score at 6 and 12 months (*n* = 223), 26% experienced an improvement in DRS score, 72% had no change, and 3% experienced a deterioration (Fig. 3). Of patients with a stable GOSE score of 3, 46% experienced up to 11 points lower DRS score (i.e. improvement) at 12 months. Of patients with a stable GOSE score of 8, 8% experienced an improvement up to 3 points lower DRS score. The change in DRS score in patients with a stable GOSE score of 8 was due to improvement in other injuries (Figure 3).

## Discussion

In this prospective cohort study, ∼60% and 30% of surviving patients with moTBI and sTBI, respectively, had a good recovery 12 months after the injury, and most reached the upper level of good recovery. An improvement in GOSE score after 6 months was observed in approximately one third of all surviving patients. The proportion that improved was lowest among patients with GOSE score of 3 at 6 months. However, approximately one fourth of the patients with seemingly stable functioning had an improvement in DRS score during the same period, typically patients with severe disability.

The proportions of patients with good recovery after mo/sTBI in our study are similar to those in most previous studies.^3,4,9,10,15–17,39–45^ However, a few studies have reported better^5,16,46,47^ or worse^15,48^ outcome. Differences in the study populations may explain some of the differences.^49^ Importantly, our study and others demonstrate that many patients with mo/sTBI do not achieve a good recovery at 12 months. There are no guidelines regarding rehabilitation after TBI, and rehabilitation needs are often unmet.^50^ Therefore, it is important that healthcare professionals are aware of the continued needs of these patients and provide follow-up over time.

A change in outcome after 6 months, most often an improvement, was observed in as much as one third of patients with mo/sTBI. Change in outcome measured with the GOSE between 6 and 12 months had not been much studied earlier. Our findings are consistent with those of two recent studies reporting an improvement in 39% and 38% of surviving patients with sTBI.^9,27^ Another study found that 62% of surviving patients with sTBI had an improvement in GOSE score from 6 to 12 months.^4^ However, that study had a younger study population, which might explain some of the difference in changes in GOSE score, in line with our finding that younger age was associated with improvement. It was further encouraging to find that most patients with a GOSE score of 8 at 6 months had the same score at 12 months, indicating an actual good function rather than a high score at 6 months resulting from over-optimism or lack of insight.

The high percentage of patients with change in GOSE score after the 6-month follow-up, even in patients with moTBI, show that the use of the 6-month end-point poses drawbacks when reporting functional outcome. Consequently, the limitations of the 6-month outcome evaluation need to be considered when interpreting the 6-month outcomes reported in large studies such as the Collaborative European NeuroTrauma Effectiveness Research in Traumatic Brain Injury (CENTER-TBI), Corticoid Randomisation After Significant Head injury (CRASH) and International Mission on Prognosis and Analysis of Clinical Trials (IMPACT).^51–53^

### Factors associated with change in GOSE score from 6 to 12 months

Notably, younger age was associated with improvement in functional outcome, after adjusting for sex, level of education, comorbidty, and GCS score. In contrast, a previous study found no association between age and improvement from 6 to 12 months in patients with sTBI.^4^ However, some of the difference may be explained by their use of the five-level GOS,^54^ which has largely been replaced by the GOSE, which was developed to improve sensitivity of the GOS.^55,56^ Further, the previous study did not exclude patients who already had the highest possible GOS score at 6 months from the analysis, and thereby there were probably many younger patients also in the “not improved” category. A previous multi-center study on sTBI found the increase in median GOSE score from 3 to 12 months to be age dependent, in accordance with our study, without significant improvement for geriatric patients.^46^ It is to be emphasized however, that patients >65 years of age received specialized in-hospital rehabilitation markedly less often than younger patients,^46^ which was also observed in our study.

In the univariable analysis, the odds of improved functional outcome after 6 months were significantly lower among patients with pre-injury disabilities. The evidence of an association was, however, weaker and the result was not statistically significant in the multivariable model, possibly because of the adjustment of age. However, a previous study found that total health burden (not including TBI) adjusted for age negatively influenced longitudinal cognitive function after mo/sTBI up to 10 years after injury.^57^ Understanding the relationship between functional outcome and comorbidity may be useful to healthcare providers tailoring rehabilitation plans to individual patient needs. This might be particularly important for older patients, who today, unfortunately, infrequently receive specialized in-hospital rehabilitation for TBI.

An important finding was that higher GCS scores in the multivariable analysis were associated with improvement in outcome, supported by another study in which improvement in GOS score from 6 to 12 months was greater for patients with GCS score 6–8 than in those with GCS score 3–5.^4^ The association between GCS score and change in outcome, emphasizes the detrimental consequences of the most severe injuries. It is, however, possible that more severely injured patients require an extended recovery period, possibly beyond 12 months following the injury, in correspondence with observations in patients with disorders of consciousness resulting from TBI.^58^

Another striking finding was that few patients with a 6 months GOSE score of 3 experienced changes in dependence in daily living from 6 to 12 months after the injury. However, they demonstrated the greatest improvement in DRS scores. A study of patients diagnosed with disorders of consciousness on arrival at rehabilitation, found that two common GOSE cut-points for dependency, ≤3 and ≤4, overestimated functional dependency in 46–61% of the participants at 12-month follow-up.^59^ These were classified as dependent by the GOSE but did not meet Functional Independence Measure-dependency criteria (score <80).^59^ Supported by previous literature, our finding shows that the GOSE categories may be insufficiently detailed to detect minor, yet meaningful, improvements in outcome, particularly in more severely injured patients. Therefore, future studies should aim to combine outcome parameters for a more complete description of functional ability.

### Study limitations

The prospective nature of this study, the large sample size, high inclusion and follow-up rates, and interviews of close relatives or caregivers, are strengths of this study. There are, however, limitations to the study. First, the study population comprised patients treated at a neurosurgical referral center; hence, the results may not be representative for patients treated only at general hospitals in the region. In a related study, we found that patients with low risk of deterioration, with a good recovery, as well as elderly patients, were commonly treated solely at the general hospitals.^60^ St. Olavs Hospital serves, however, as a general hospital for a substantial part of the catchment area. Second, the GOSE was scored in relation to TBI, whereas the DRS was scored with respect to all diseases/disabilities. Such a discrepancy was, however, mainly present in patients able to return to previous life roles (GOSE scores 7 and 8), who were on sick leave at 6 months because of, for example, major orthopedic trauma (DRS 3). Lastly, the long inclusion period is another limitation of this study. However, in a region with a low incidence rate of mo/sTBI,^12^ a long inclusion period is critical to obtain a large sample size.

## Conclusion

Good recovery was observed in as much as two thirds of moTBI, whereas after sTBI, moderate to severe disability was the most frequent outcome at 12 months. Most patients with good recovery reached the upper level, whereas most patients with severe disability were at the lower level. Change, mostly improvement, in GOSE score beyond 6 months was quite common, arguing against the use of 6-month outcome as a time end-point in research. The GOSE appears to lack sensitivity to small changes in functioning, particularly in the lower categories of the scale. Therefore, a combination of outcome measures needs to be considered to comprehensively capture the consequences after TBI.

## Abbreviations Used

CENTER-TBI: Collaborative European NeuroTrauma Effectiveness Research in Traumatic Brain Injury
CI: confidence interval
CRASH: Corticoid Randomisation After Significant Head injury
CT: computed tomography
DRS: Disability Rating Scale
GCS: Glasgow Coma Scale
GOSE: Glasgow Outcome Scale Extended
IMPACT: International Mission on Prognosis and Analysis of Clinical Trials
moTBI: moderate traumatic brain injury
OR: odds ratio
sTBI: severe traumatic brain injury
TBI: traumatic brain injury
